# Patterns of Cervical Lymph Node Metastases in Primary and Recurrent Papillary Thyroid Cancer

**DOI:** 10.1155/2011/735678

**Published:** 2011-11-17

**Authors:** Neda Ahmadi, Ameet Grewal, Bruce J. Davidson

**Affiliations:** Department of Otolaryngology-Head and Neck Surgery, Georgetown University Hospital, Washington, DC 20007, USA

## Abstract

The incidence of thyroid cancer is rising in the United States with papillary thyroid cancer (PTC) being the most common type. We performed a retrospective study of 49 patients with PTC who underwent 57 lateral neck dissections (NDs). The extent of NDs varied, but 29 of 57 (51%) consisted of levels II–V. Twelve of 57 (21%) NDs consisted of levels I–V. Twelve of 57 (21%) NDs consisted of levels II–IV. One of 57 (1.8%) necks involved only levels I–IV. One of 57(1.8%) necks involved only levels I–V. One of 57(1.8%) necks involved only levels III–V. Two (3.5%) double-level (III–IV) neck surgeries were also performed. Metastatic PTC adenopathy was confirmed pathologically in 2%-level-I, 45%-level-II, 57%-level-III, 60%-level-IV, and 22%-level-V necks. Level-V was positive in 21% of primary and 24% of recurrent groups (*P* = 0.76). Comparing primary and recurrent disease, there was no difference in nodal distribution or frequency for levels I, II, III, and V. Level-IV was more common in the recurrent cases (*P* = 0.05). Based on the pathologic distribution of nodes, dissection should routinely include levels II–IV and extend to level-V in primary and recurrent cases. Our data does not suggest routine dissection of level-I.

## 1. Introduction

The incidence of thyroid cancer is rapidly rising in the United States at a rate of 4% per year for the past twenty years [1]. In 2010, there were 44,670 newly diagnosed cases and 1,690 deaths reported from thyroid cancer [2]. 

Papillary thyroid cancer (PTC) is the most common type of thyroid cancer. However, despite its indolent course, it accounts for more than 50% of deaths from thyroid cancer [3, 4]. In 1998, the National Cancer Database reported on a series of 53,856 patients with thyroid cancer from 1985 to 1995 in the United States. In this series, PTC demonstrated an overall 10-year survival rate of 99% [5]. Despite this excellent survival, PTC is associated with a high rate (30% to 90% of patients) of overall lymph node metastases [6]. This has led to controversy regarding the optimal surgical management of the neck in thyroid cancer. Despite this controversy, recent data indicates that volume of neck dissections performed in the United States for thyroid and parathyroid diseases increased from 2,822 in 2000 to 5,282 in 2006 [7].

Previous studies have reported on the patterns of cervical lymph node metastases and have made recommendations regarding the neck treatment [8–11]. The American Thyroid Association (ATA) recommends preoperative cervical (central and lateral) lymph node ultrasound (US) on all patients with biopsy-proven thyroid malignancy and fine needle aspiration of all sonographically suspicious (loss of fatty hilus, rounded shape, hypoechogencity, cystic change, calcification, and peripheral vascularity) lymph nodes. Lateral neck dissection is recommended for patients with biopsy-proven metastatic lymphadenopathy. Although the ATA favors “en bloc” neck dissection over “berry picking,” they do not make specific recommendations regarding which neck levels should be operated on [12]. 

The impact of neck dissection on overall survival is unclear [8, 10, 13]. Lateral neck dissection has been shown to afford a survival advantage in certain subsets of patients. For instance, data from Japan has indicated a survival advantage for neck dissection in patients with gross nodal involvement, in women older than 60 years, and when the primary tumor extends beyond the thyroid capsule [10]. It is suggested that when lateral neck metastases are identified, neck dissection provides good regional control, improves the efficacy of radioactive iodine ablation of microscopic disease, and allows for a more accurate monitoring of posttreatment serum thyroglobulin levels [8]. 

There are many unanswered questions when it comes to the management of lateral neck metastases in PTC. While radical neck dissection is rarely performed for this disease, what extent of neck dissection is appropriate? Which neck levels should be included in these neck dissections? What are the patterns of lateral lymph node metastases in primary versus recurrent cases of PTC? The objective of this retrospective study is to present our data in order to better understand the patterns of lateral lymph node metastases in patients with primary and recurrent PTC and to evaluate outcomes in these patients.

## 2. Material and Methods

We reviewed the medical records of a series of patients who underwent lateral neck dissection (LND) for PTC at Georgetown University Hospital (GUH), Washington, DC, Department of Otolaryngology-Head and Neck Surgery between 1995 and 2009. Institutional review board approval was obtained for this study. All of the patients had pathologic diagnosis of PTC. Data regarding demographics, prior history of thyroid cancer, prior treatments (including surgical and radioactive iodine), extent of lymphadenectomy, and total number of nodes removed were collected. Patients who had not received any prior thyroid treatment (surgery with or without radioactive iodine) were categorized as the primary group, and patients who had received prior treatment (thyroidectomy with or without radioactive iodine) for PTC were categorized as the recurrent group. The pathology reports were analyzed to determine the incidence of metastatic disease at each level of the neck. We also gathered information on postsurgical and postradioactive iodine serum thyroglobulin levels, neck control, followup, and current disease status. Neck control was defined as the absence of clinical, pathologic, and imaging evidence of recurrence. Where data allowed, outcomes were also assessed relative to thyroglobulin levels before and after neck dissection.

Patients underwent LND either during the initial thyroidectomy or at a later date when recurrent disease in the lateral neck was noted. In all cases, lateral lymphadenopathy was detected based on preoperative radiographic or clinical examination. In the case of recurrent disease, none of the patients underwent LND for serum thyroglobulin (Tg) elevation only. All of the patients had confirmed pathologic involvement at neck dissection. Neck levels were considered positive for disease if they had at least one pathologically metastatic node. 

Analyses were performed comparing the proportion of neck levels documented to contain positive disease. Chi-square and Fisher's exact tests were performed in order to compare nodal positivity in the different neck levels between the primary and recurrent groups. A *P* value of <0.05 was considered significant.

## 3. Results

### 3.1. Patient Information

Forty-nine patients were identified: 27 (55%) women and 22 (45%) men. The mean age in this study was 41 (14–69) years. Four (8%) had a family history of differentiated thyroid cancer. 

Twenty-five patients were treated for primary disease, and 28 patients were treated for recurrent disease. Twenty-three of the 28 (82%) patients in the recurrent group were previously treated for PTC at outside institutions prior to presenting to GUH. The other five patients were treated for both primary and recurrent disease at GUH. 

All of the patients in the recurrent group had undergone surgical treatment, and 26 (93%) had also received radioactive iodine treatment (Table 1). The cumulative radioactive iodine dose prior to neck dissection in this group ranged from 96 mCi to 743 mCi.

### 3.2. Lateral Neck Dissection

A total of 57 neck dissections were performed on 49 patients. Four (8.2%) patients underwent simultaneous bilateral neck dissections. The extent of neck dissections varied, but 29 of 57 (51%) consisted of levels II–V. Twelve of 57 (21%) neck dissections consisted of levels I–V. Twelve of 57 (21%) neck dissections consisted of levels II–IV. One of 57 (1.8%) necks involved only levels I–IV. One of 57 (1.8%) necks involved only levels I–V. One of 57 (1.8%) necks involved only levels III–V. Two (3.5%) double-level (III-IV) neck surgeries were also performed. One of these was performed on the contralateral neck in a GUH patient undergoing bilateral neck dissections. One of these was performed on a patient who was previously treated at an OSH with total thyroidectomy only. Overall, neck dissections included the following levels: 14 of 57 (25%) included level I, 55 of 57 (96%) included level II, 57 of 57 (100%) included level III, 57 of 57 (100%) included level IV, and 43 of 57 (75%) included level V. 

An average of 31 (4 to 94) lymph nodes were removed with each neck dissection specimen. An average of 7 lymph nodes were positive with each neck specimen with a range of 1 to 94 nodes. Among all of the patients treated, metastatic PTC adenopathy was confirmed pathologically in 2% of level I, 45% of level II, 57% of level III, 60% of level IV, and 22% of level V neck levels. When comparing primary and recurrent cases (Table 2), levels II, III, and IV were the most common and level I was the least commonly involved in both groups. Comparing primary and recurrent cases, there was no difference in the prevalence of positive disease at levels I, II, and III. Level IV was more common in the recurrent cases (*P* = 0.05). Level V involvement was equivalent in the recurrent (24%) and in the primary (21%) cases (*P* = 0.76).

### 3.3. Central Neck Dissection

Central neck dissections (CNDs) were performed in 21 of 25 patients in the primary group. An average of 8 (0 to 29) lymph nodes were removed with each neck specimen. An average of 5 (0 to 25) lymph nodes were positive with each neck specimen. Among all of the necks treated in this group, metastatic PTC adenopathy to the medial compartment was confirmed pathologically in 19 of 21 (90%) necks. Two of 21 (9.5%) patients in this group had pathologically positive lateral neck nodes, but did not have positive level VI nodes. One of these patients had positive lateral neck nodes in levels II and III, and one patient had positive lateral neck nodes in levels II–V.

CNDs were performed in 5 of 28 patients in the recurrent group. All of these were performed in patients who had their primary treatment at an outside institution prior to presenting to GUH. CNDs were not performed as a part of their primary treatment. An average of 5 (0 to 18) lymph nodes were removed with each neck specimen. An average of 2 (0 to 8) lymph nodes were positive with each neck specimen. Metastatic PTC adenopathy to the medial compartment was confirmed pathologically in 3 of 5 (60%) necks.

### 3.4. Radioactive Iodine following Neck Dissection

Twenty-four of 25 (96%) patients in the primary group received radioactive iodine (I-131). The cumulative dose of I-131 following neck dissection ranged from 102 mCi to 650 mCi. Twelve of 28 (43%) patients in the recurrent group received I-131 following neck dissection. The cumulative dose of I-131 following neck dissection in this group ranged from 150 mCi to 507 mCi.

### 3.5. Clinical Control

Follow-up information was available on 43 of 49 (88%) patients. At a median of 34 (1–111) months, the follow-up status was as follows: 38 of 43 patients (88%) alive without evidence of clinical disease, 4 of 43 patients (9%) alive with clinical disease, and 1 patient (2%) deceased from metastatic thyroid cancer. Of the 4 patients who are alive with disease at last followup, 1 will be treated with external beam radiation therapy for a solitary neck recurrence, one has recurrence in the neck and mediastinum, and 2 patients have distant metastases. 

Follow-up information was available in 51 of 57 (89%) necks. Pathologic control of disease in the operated neck was seen in 48 of 51 (94%) necks at a median followup of 34 (1–111) months. Three (6%) patients had ipsilateral neck recurrences. Of these, 1 patient recurred in a previously unoperated level I. One patient recurred in a previously operated level III, and another patient recurred in a previously operated level IV. Overall, there were only 2 (3.9%) recurrences within previously operated neck levels.

### 3.6. Thyroglobulin Control

Following neck dissection and radioactive iodine adminstration serum thyroglobulin levels with thyroid stimulating hormone (TSH) suppression and stimulation were analyzed. In the TSH suppression data, 4 of 49 (8.2%) patients had positive thyroglobulin antibodies and were eliminated. TSH-suppressed thyroglobulin information was available in 42 of 45 (93%) patients. Thyroglobulin level was <1.0 ng/mL in 27 of 42 (64%) patients at a median followup of 14 (1–76) months. In the rhTSH stimulation data, 4 of 49 (8.2%) patients had positive thyroglobulin antibodies and were eliminated. TSH-stimulated thyroglobulin information was available in 32 of 45 (71%) patients. Stimulated thyroglobulin level was <1.0 ng/mL in 15 of 32 (47%) patients at a median followup of 15 months (1.5–120). 

We also analyzed the number of cases with undetectable thyroglobulin levels. Undetectable thyroglobulin level was designated as the lowest range reported by the laboratory (either <0.5 ng/mL or <0.2 ng/mL depending upon the laboratory). In the primary group, undetectable thyroglobulin levels were present in 9/16 (56%) under TSH suppression and in 6/14 (43%) patients with rhTSH stimulation. In the recurrent group, undetectable thyroglobulin levels were present in 15/26 (58%) of patients under TSH suppression and 5/18 (27%) with rhTSH stimulation.

## 4. Discussion

In the present series, LND was performed to address suspicious lymphadenopathy that was evident on clinical examination, imaging, or intraoperatively. We found no differences in nodal distribution between primary and recurrent cases. Clinical control in the operated neck is excellent in both primary and recurrent cases. The majority of primary patients and a substantial minority of patients with recurrence in the neck demonstrated undetectable stimulated Tg levels in followup. 

Sivanandan and Soo [14] described the most commonly involved neck levels in PTC. In their study of 75 patients with primary PTC, levels II–IV were frequently involved, with level III being the most common site for lateral neck metastases. Kupferman et al. [8] described similar results in their study of 39 patients (44 neck dissections). The distribution of nodal positivity in levels I, II, III, IV, and V in this study was 14%, 52%, 57%, 41%, and 21%, respectively. As demonstrated in Table 3, other studies have also demonstrated that levels II–IV are the most frequently involved cervical nodal basins [9, 15, 16]. Our study adds support to this previous data with metastatic PTC commonly present in levels II (45%), III (57%), and IV (60%). 

When comparing primary and recurrent groups, there were no significant differences in the rate of positive disease in levels II-III. Level IV was more common in recurrent cases, which was significant. Despite this, due to the high rates of nodal positivity (Table 2), we would recommend performing routine level IV dissection in both primary and recurrent cases. 

Overall, level I involvement (2%) was rare; this is comparable to other studies [8, 14]. Level I nodal metastases were more common but not statistically significant in the recurrent cases (3.4%). No level I involvement was noted in the primary cases. Similar to Sivanandan and Soo [14], level I was never involved in isolation. Given the low rate of level I metastases in both primary and recurrent cases, we do not advocate routine dissection of level I unless exam, imaging, or biopsy indicates involvement. 

It is important to determine whether level V lymphadenectomy is necessary in PTC because this portion of the procedure can be associated with postoperative morbidity related to the spinal accessory nerve (SAN). Among the necks treated, metastatic adenopathy was confirmed pathologically in 22% of level V. This is similar to the 21% and 29% incidences described by others, but is lower than the 40% incidence reported by Kupferman et al. [8], Sivanandan and Soo [14], and Farrag et al. [18]. Overall, previous studies have shown that level V harbors metastatic disease in 21% to 60% of necks [8, 17]. In our series, when the primary and recurrent cases were analyzed, 21% and 24% of the necks harbored metastatic PTC, respectively. Level V was never involved with disease in isolation. 

Farrag et al. [18] divide level V-A and V-B based on the horizontal plane marking the inferior border of the cricoid cartilage. They recommend dissection of level V-B only in order to minimize the risk of damage to the SAN. In our practice, dissection of level V typically includes localization and dissection of a portion of level V-A and all of V-B by dissecting along, but not posterior to; the SAN. We believe that this provides the safety of nerve identification while avoiding circumferential mobilization of the nerve. We suspect that this will reduce the risk of SAN-associated morbidity including shoulder pain and dysfunction while allowing an adequate dissection of level V. In our practice, dissection of the portion of level V above the spinal accessory nerve is based upon imaging and intraoperative findings.

We agree with the ATA recommendations to perform CND in patients with PTC who have clinically involved central neck lymphadenopathy. In patients with clinically uninvolved central neck lymphadenopathy, we also agree with the ATA guidelines to perform elective CND for advanced primary tumors (T3 or T4) [12]. Therefore, in primary cases with lateral neck disease, we recommend CND in all cases, and in recurrent cases with lateral neck disease, we recommend careful imaging and intraoperative evaluation of the central neck and low threshold for CND. In these recurrent cases, if there is no suspicion of central neck disease, surgical judgment must consider preoperative parathyroid status, recurrent nerve function, and overall disease burden before pursuing elective central neck dissection. 

Pathologic neck control in our study was excellent (94%). Only two patients developed recurrences in a previously operated neck level resulting in a 96% neck control within previously operated neck levels. 

TSH-stimulated undetectable Tg levels were identified in 43% and 27% of our primary and recurrent patients after median followup of 16 (2–88) and 14 (1–98) months, respectively. In a study of recurrent lateral neck disease by Al-Saif et al. [4], TSH-stimulated Tg levels were undetectable in 24% of patients with recurrent PTC after a five-year followup. We recognize that our study is limited by a shorter duration of followup.

## 5. Conclusions

We recognize that our study is limited by insufficient follow up data on some of our patients. However, we believe that our study gives important insight into the management of lateral lymph node metastases in patients with primary and recurrent PTC. Neck dissection in these cases should include levels II–IV. Level I dissection is not necessary in primary or recurrent cases unless exam or imaging indicates involvement. Our data suggests performing dissection of level V in both primary and recurrent cases. The extent of level V dissection required remains an area for further study.

## Figures and Tables

**Table 1:** Surgical and nonsurgical treatments of patients in the recurrent group prior to undergoing a secondary lateral neck dissection.

	*N* = 28 (%)
Partial thyroidectomy	1 (3.6%)
Total thyroidectomy	9 (32%)
Subtotal thyroidectomy + Level VI	1 (3.6%)
Total thyroidectomy + Level VI	8 (28.6%)
Total thyroidectomy + LND*	
Ipsilateral**	1 (3.6%)
Contralateral^+^	3 (10.7%)
Total thyroidectomy + LND*+ Level VI	
Ipsilateral**	1 (3.6%)
Contralateral^+^	2 (7.1%)
Radioactive iodine (I-131)	26 (93%)

*Lateral neck dissection.

^+^Of the 5 patients who had previously undergone a contralateral neck dissection, 3 were performed at GUH and 2 were performed at outside institutions.

**Of the 2 patients who had previously undergone an ipsilateral neck dissection, both were performed at outside institutions.

**Table 2:** Comparison of nodal positivity in the primary and recurrent cases. *n* represents the number of overall specific neck levels dissected.

Neck level (*n*)	Primary group	Recurrent group	*P* value
I (14)	0%	3.4%	0.62
II (55)	50%	41%	0.30
III (57)	57%	62%	0.87
IV (57)	46%	76%	0.05
V (43)	21%	24%	0.76

**Table 3:** Nodal positivity in patients with PTC within different levels of the neck.

	*N*	I	II	III	IV	V	VI
Sivanandan and Soo [14]	75	3.75%	48.7%	65%	56.3%	28.7%	—
Pingpank et al. [15]	44	37.5%	43.1%	76.4%	58.8%	28%	—
Kupferman et al. [8]	39	14%	52%	57%	41%	21%	—
Lee et al. [16]	167	—	55.5%	80.6%	74.9%	16.8%	—
Roh et al. [9]	52	3.7%	72.2%	72.2%	75.9%	12.9%	84.6%
Kupferman et al. [17]	70	27%	57%	62%	62%	53%	77%
Farrag et al. [18]	53	—	60%	66%	50%	40%	—
